# Prevalence and Prognostic Significance of Chloride Levels in Patients with Acute Medical Conditions: A Prospective Observational Study

**DOI:** 10.3390/life15040676

**Published:** 2025-04-21

**Authors:** Rhita Bennis Nechba, Jihane Belayachi, Mina Agrou, Elmostapha El Fahime, Nawal Meknassi, Maha Louriz, Naoufel Madani, Redouane Abouqal

**Affiliations:** 1 Acute Medical Unit, Ibn Sina University Hospital, Rabat 10000, Morocco; bennirhita@yahoo.fr (R.B.N.); jihanebelayachi@gmail.com (J.B.); mina.agrou@gmail.com (M.A.); nawalmeknassi19@gmail.com (N.M.); maariz@gmail.com (M.L.); naoufelmad@gmail.com (N.M.); 2 Laboratory of Biostatistics, Clinicial, and Epidemiological Research, Faculty of Medecine and Pharmacy, Mohammed V University of Rabat, Rabat 10000, Morocco; 3 Laboratory of Physiology, Faculty of Medecine and Pharmacy, Mohammed V University of Rabat, Rabat 10000, Morocco; 4 Molecular Biology and Functional Genomics Platform, National Center for Scientific and Technical Research (CNRST), Rabat 10102, Morocco; elfahime@cnrst.ma

**Keywords:** prevalence, prognosis, chloride, prospective observational study

## Abstract

Chloride plays a considerable role in physiology. This study aimed to assess the association between serum chloride and prognosis in the population of adults with acute medical conditions. A prospective cohort study was conducted in an acute medical unit. Chloride levels at admission were the main exposure factor, categorized into hypochloremia, normochloremia, and hyperchloremia. The outcomes were in-hospital mortality and length of hospital stay (LOHS). A total of 798 patients were included. The mean age was 57.3 ± 18.3 years. The prevalence of dyschloremia was 40.9%. Restricted cubic splines revealed a linear association between hypochloremia and in-hospital mortality, as well as between hypochloremia and LOHS. After adjusting for age, sex, heart failure, diabetes, sodium, bicarbonates, creatinine, and diuretic use, hypochloremia was significantly associated with in-hospital mortality (OR = 2.23; 95% CI: 1.29, 3.86, *p* = 0.006), but not hyperchloremia (*p* = 0.57). Similarly, it was associated with a longer LOHS (β = 2.19; 95% CI: 0.01, 4.39, *p* = 0.05), but not hyperchloremia (*p* = 0.8). The interaction between chloride and sodium levels was not significant (*p* = 0.61). Subgroup analysis showed that the effect of hypochloremia on in-hospital mortality was consistent across subgroups. The prevalence of dyschloremia in this study was high at 40.9%. Hypochloremia increased the risk of in-hospital mortality and extended the LOHS. Differentiating the effects of chloride levels from those of sodium can enhance clinical risk stratification and enable a more targeted management approach for acutely ill patients. Recognizing this distinction is essential for optimizing prognostic assessment and tailoring treatment strategies accordingly.

## 1. Introduction

Chloride is the principal extracellular anion and plays a significant role in physiology [1,2]. It is involved in acid–base balance, osmolarity, electroneutrality of body fluids, immune regulation, and muscle activity [1,2,3]. Various mechanisms and hormones regulate its concentration, including the renin–angiotensin–aldosterone system, the atrial natriuretic peptide, the sympathetic nervous system, and other factors affecting renal blood flow and renal salt reabsorption [4,5]. Chloride’s own physiology is certainly not yet fully elucidated. Dyschloremias are common in critically ill patients [6]. Despite the physiological importance of chloride, its prognostic role has long been neglected. Several factors can be implicated in the occurrence of hypochloremia in these patients, such as renal losses mainly related to the use of diuretics, digestive losses, chronic respiratory acidosis, heart failure, inappropriate secretion of antidiuretic hormone, and excessive infusion of hypotonic solutions [1,4,7]. Hyperchloremia, on the other hand, is due to a loss of pure water associated with hypernatremia, and this occurs through the skin during febrile syndromes, burns, excessive sweating, thyrotoxicosis, and hypermetabolism. Water loss can also be of digestive or renal origin [6]. On the other hand, hyperchloremia can also be of iatrogenic origin during massive vascular fillings with chloride-rich solutions [6,8]. In recent years, dyschloremias have attracted increasing attention, especially in specific populations. Previous studies have evaluated the prognostic significance of dyschloremias in acute heart failure patients [9,10,11], chronic heart failure patients [12,13], coronary patients [14], patients suffering from pulmonary arterial hypertension, and those in acute cirrhotic decompensation [15]. Few studies have been conducted on a large population of critically ill patients [2,16,17]. This study aimed to assess the association between serum chloride and prognosis in the population of adults with acute medical conditions.

## 2. Materials and Methods

### 2.1. Study Design

This was a prospective cohort study conducted in the Acute Medical Unit (AMU) of the Ibn Sina University Hospital Center in Rabat, Morocco. This hospital serves residents of northwestern Morocco. It is a tertiary hospital with 1028 beds and has been operational since 1955. The bed occupancy rate ranges between 76% and 85%. The hospital comprises 24 departments (12 surgical units, 9 medical units, and 3 intensive care units) and admits adult patients; those requiring gynecology-obstetrics and pediatrics specialties are treated in other facilities. The AMU is a unit that manages patients with urgent medical conditions requiring hospitalization when no beds are available in the relevant hospital department; patients with intermediate clinical severity between intensive care and conventional care units; and patients with complex medical conditions and multiple comorbidities for whom it has not been possible to determine a specific department for care. All patients are admitted from the emergency medical unit. The unit accommodates approximately 1200 patients per year with an average age of 40 years. It comprises seven individual rooms and four shared rooms (six beds per room). The average length of stay is 5 days.

### 2.2. Data Source

This study adhered to the ethical guidelines set forth in the Declaration of Helsinki [18]. Approval was obtained from the Ethics Committee for Biomedical Research of the Mohammed V University of Rabat, reference number: IQRG0006594. Informed written consent for participation was obtained from all subjects involved in the study. The study duration was 7 months, from January to July 2019. Patient data were prospectively extracted from patient records to minimize information bias. Survival data were collected by researchers involved in the study, who obtained information either by directly contacting patients and their physicians at the hospital or through interviews with their families. The guidelines for ensuring well-conducted observational studies in epidemiology were followed in this work [19].

### 2.3. Participants

All patients admitted to the unit with serum chloride levels at admission were included. Patients admitted without serum chloride levels at admission were excluded.

### 2.4. Collected Variables

Data for each patient were collected within the first 24 h following their admission to the AMU. This timeframe included initial assessments conducted in the emergency room at the time of admission, as well as subsequent evaluations performed in the AMU. The main variable was the chloride level in mmol/L. It was studied in continuous form and categorized into three groups based on the serum chloride level: group 1, hypochloremia with a serum chloride < 98 mmol/L; group 2, normochloremia, with a serum chloride level between 98–106 mmol/L; and group 3, hyperchloremia, with a serum chloride level > 106 mmol/L.

Several covariables were collected: demographic, clinical, biological, and therapeutic. Demographic data included age (years) and sex (male, female). Comorbidities were classified into six categories: chronic diseases, arterial hypertension, heart failure, diabetes mellitus, chronic kidney failure, and cancer. The clinical characteristics collected were Charlson score [20], mean arterial pressure (mmHg), heart rate (beats per minute), respiratory rate (cycles per minute), and temperature (degrees Celsius). Laboratory measurements included a blood ionogram with chloride (mmol/L), sodium (mmol/L), bicarbonate (mmol/L), urea (mmol/L), and creatinine (μmol/L). We also collected the use of diuretics data.

### 2.5. Outcome Criteria

The primary outcome criterion was hospital mortality. The secondary outcome criterion was the length of hospital stay (LOHS).

### 2.6. Statistical Analysis

First, we conducted descriptive statistical studies. Quantitative variables were expressed as mean ± standard deviation or median and interquartile range, and qualitative variables as number and percentage. Then, a univariate analysis was performed where the variables were compared using the ANOVA test, Kruskal–Wallis test, and χ^2^ test. To compare the characteristics of study participants across different serum chloride groups (hypochloremia, normochloremia, and hyperchloremia), we used analysis of variance (ANOVA) for normally distributed continuous variables and the Kruskal–Wallis test for non-normally distributed variables. The normality of each dataset was assessed using the Shapiro–Wilk test. If the data met the assumptions of normality, we applied parametric tests; otherwise, non-parametric methods were used to ensure the accuracy and validity of the results. For post hoc analysis, following ANOVA, Tukey’s test was conducted to identify significant differences between the individual groups. This test was chosen because it allows for pairwise comparisons while controlling for the type I error rate across multiple comparisons. For non-normally distributed data, pairwise comparisons were performed using the Mann–Whitney U test with appropriate corrections. Next, multivariable statistical analyses were conducted with predefined adjustment variables: namely, age, gender, the presence of heart failure, diabetes mellitus, serum sodium level, serum bicarbonate level, serum creatinine level, and the use of diuretics. A logistic regression model was conducted to evaluate the relationship between serum chloride levels and hospital mortality. A multivariate linear regression model was conducted to evaluate the relationship between serum chloride levels and the LOHS. The models examining the relationship between chloremia and hospital mortality were studied using fractional polynomial interactions and plotted at different values of natremia (105, 115, 125, 135, 145, 155, 165) (mmol/L) with adjustments for age, sex, the presence of heart failure, diabetes mellitus, serum sodium level, serum bicarbonate level, serum creatinine level, and the use of diuretics. Similarly, we graphically represented restricted cubic spline curves to study a potential nonlinear association between chloremia and mortality on one hand, and between chloremia and LOHS on the other. Subgroup analysis was then performed after stratification with predefined relevant covariates. We used Stata Release 14 and R 4.1.3 software for statistical analysis. A *p* value < 0.05 was considered significant for all main effects, and a *p* value < 0.1 was considered significant for interactions.

### 2.7. Results

The number of patients included in this study was determined by specific inclusion criteria and study duration constraints. In fact, data collection was interrupted during the COVID-19 pandemic due to significant changes in patient profiles and clinical priorities. A total of 798 patients with a mean age of 57.3 ± 18.6 years were included in the study; 36.8% were over 65 years old, 63.8% had chronic diseases, and 17.3% used diuretics. Table 1 shows the sociodemographics and clinical characteristics of the patients.

The mean chloride level was 101 ± 6.9 mmol/L, the LOHS was 8 days [5–14], and the hospital mortality rate was 12.8%. Table 2 summarizes the biological characteristics and the evolutionary criteria of the patients.

The prevalence of dyschloremia was 40.9% (*n* = 327); hypochloremia was 26.9% (*n* = 215); normochloremia was 59% (*n* = 471); and hyperchloremia was 14% (*n* = 112). In univariate analysis, the hospital mortality rate was 21.4% (*n* = 46) in the hypochloremia group, 10% (*n* = 47) in the normochloremia group, and 8% (*n* = 9) in the hyperchloremia group, *p* < 0.001. The LOHS was 9 days [6–17] in the hypochloremia group, 8 days [5–13] in the normochloremia group, and 7 days [4–13] in the hyperchloremia group, *p* = 0.012. Table 3 presents the characteristics of patients according to chloride levels at admission.

Three models were evaluated to assess the association between electrolytes and mortality. The chloride-based model showed a significant association (OR = 0.9, 95% CI: 0.90–0.96, *p* < 0.001), with an AUC of 0.636. The bicarbonate-based model showed no significant association (OR = 0.978, 95% CI: 0.94–1.01, *p* = 0.221), with an AUC of 0.581. In the combined model (chloride + bicarbonate), both variables were significantly associated with mortality (chloride: OR = 0.9, 95% CI: 0.88–0.94, *p* < 0.001; bicarbonate: OR = 0.9, 95% CI: 0.91–0.98, *p* = 0.011), with an AUC of 0.674.

In multivariable analyses and after adjusting for age, sex, the presence of heart failure, diabetes mellitus, serum sodium level, serum bicarbonate level, serum creatinine level, and the use of diuretics, logistic regression showed that hypochloremia was significantly associated with mortality (OR = 2.23; 95% CI: 1.29; 3.86, *p* = 0.006), but hyperchloremia was not (OR = 0.79; 95% CI: 0.35; 1.76, *p* = 0.57). Similarly, in multiple linear regression, hypochloremia was significantly associated with a longer LOHS (β = 2.19; 95% CI: 0.01; 4.39, *p* = 0.05), but hyperchloremia was not (β = −0.34; 95% CI: −2.94; 2.27, *p* = 0.8). Table 4 summarizes the results of the multivariable analysis.

The table presents odds ratios (OR) with 95% confidence intervals (CI) and *p*-values for in-hospital mortality, as well as beta coefficients (β) with 95% CI and *p*-values for hospital length of stay. Models were adjusted for age, sex, the presence of heart failure, diabetes mellitus, serum sodium level, serum bicarbonate level, serum creatinine level, and the use of diuretics. All coefficients and *p*-values are compared to the reference category, defined as normal chloride levels (98–106 mmol/L).

**Figure 1:**
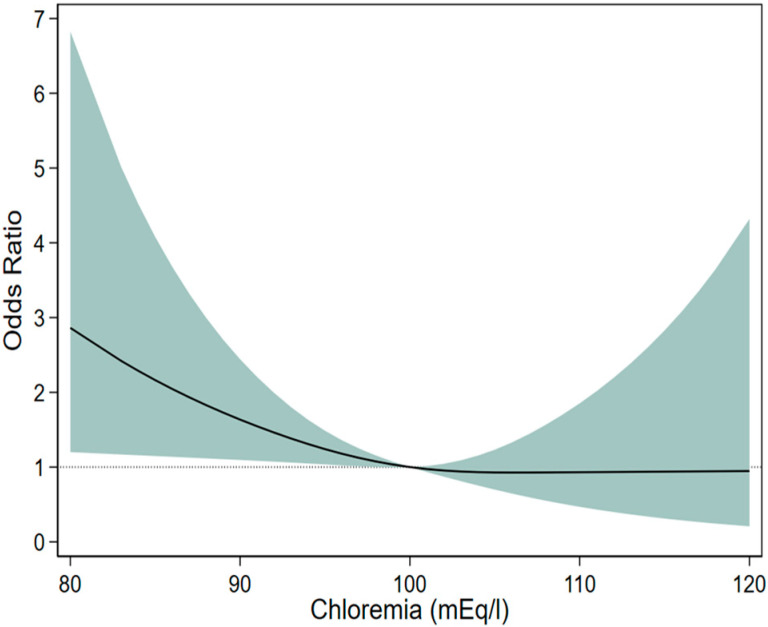
Restricted cubic spline curve showing the association between chloremia and mortality using multivariable logistic regression models. The black line represents the estimated odds ratio, and the shaded area indicates the 95% confidence interval. Each stratification was adjusted for age, sex, the presence of heart failure, diabetes mellitus, serum sodium level, serum bicarbonate level, serum creatinine level, and the use of diuretics. Legend: The adjusted odds ratios for mortality as a function of serum chloride levels (chloremia), estimated using multivariable logistic regression with restricted cubic splines. Serum chloride was modeled as a continuous variable, with a reference value set at 100 mEq/L (OR = 1) to represent baseline risk. The model is adjusted for age, sex, heart failure, diabetes mellitus, blood sodium, blood bicarbonate, blood creatinine, and diuretic use. The solid curve shows the point estimates of the odds ratios, and the shaded area represents the corresponding 95% confidence intervals, which widen at the extreme ends of the chloride distribution due to smaller sample sizes.

Restricted cubic spline curve analysis revealed in logistic regression that patients with hypochloremia had a significantly higher risk of hospital mortality, whereas those with hyperchloremia did not. These results are shown in Figure 1.

**Figure 2:**
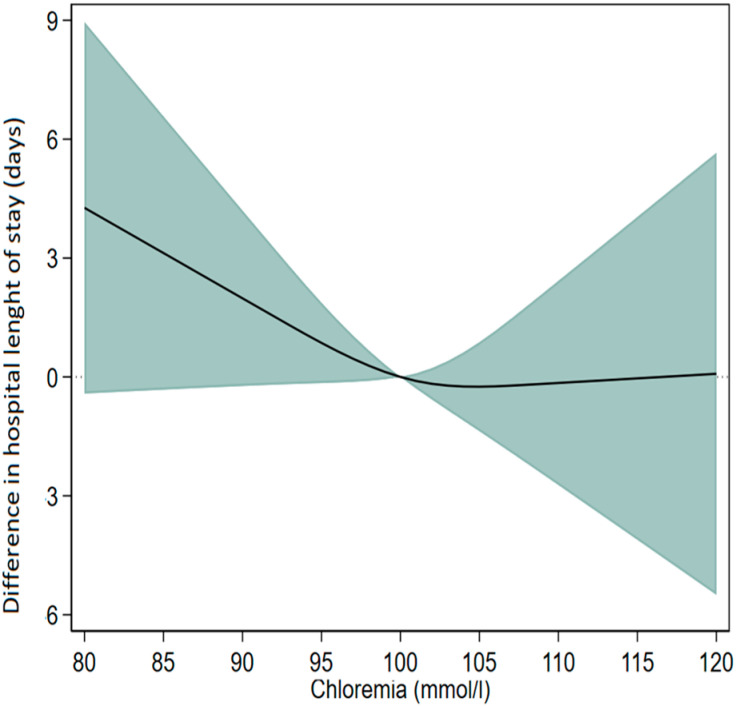
Restricted cubic spline curve showing the association between chloremia and length of hospital stay using multiple linear regression models. Each stratification was adjusted for age, sex, the presence of heart failure, diabetes mellitus, serum sodium level, serum bicarbonate level, serum creatinine level, and the use of diuretics. Legend: The restricted cubic spline curve showing the association between chloremia and length of hospital stay using multiple linear regression models. The data were adjusted for age, sex, the presence of heart failure, diabetes mellitus, serum sodium level, serum bicarbonate level, serum creatinine level, and the use of diuretics. The difference in hospital length of stay (LOHS) relative to normochloremic patients (~100 mmol/L). A value of zero represents no difference in LOHS. Positive values indicate a longer hospital stay for patients with hypochloremia, while negative values indicate a shorter stay for those with hyperchloremia. The shaded region represents the 95% confidence interval. The curve and the shaded areas around it represent the estimated values and their corresponding 95% confidence interval.

The restricted cubic spline curve also revealed in linear regression that patients with hypochloremia stayed significantly longer in the hospital, but those with hyperchloremia dd not. These results are shown in Figure 2.

**Figure 3:**
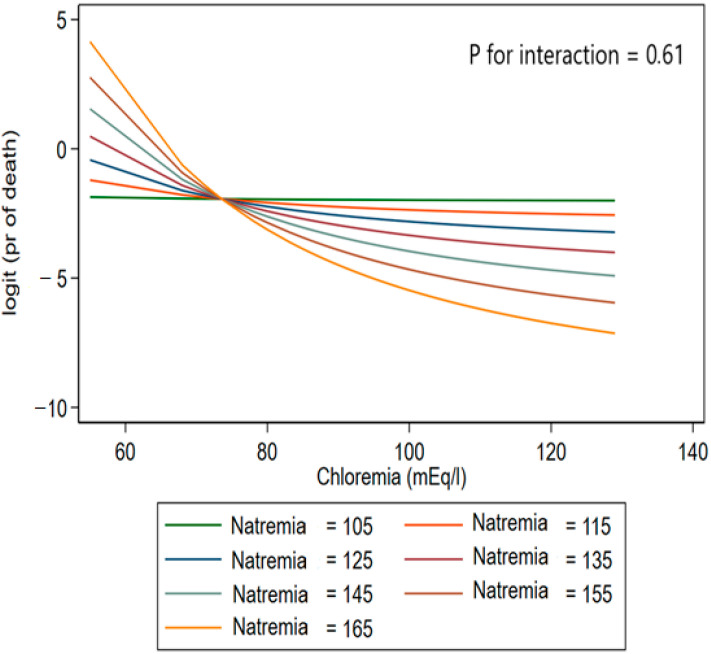
Non-linear interaction: Chloremia-Natremia: relationship between in-hospital mortality and chloremia at different natremia levels. Each stratification was adjusted for age, sex, the presence of heart failure, diabetes mellitus, serum sodium level, serum bicarbonate level, serum creatinine level, and the use of diuretics. Legend: Using fractional polynomial interactions, models of the relationship between chloremia and in-hospital mortality were plotted at different point values of natremia (105, 115, 125, 135, 145, 155, 165 mmol/L) and adjusted for age, sex, the presence of heart failure, diabetes mellitus, serum sodium level, serum bicarbonate level, serum creatinine level, and the use of diuretics (*p* = 0.61 for the interaction). While extreme values of natremia (e.g., 105 and 165 mmol/L) are rare and typically associated with critical illness, they were included for illustrative purposes to visualize interaction trends across the full range.

Using fractional polynomial interactions, hypochloremia had a strong prognostic value regardless of natremia. There was no interaction regardless of the natremia value (*p* = 0.61). These results are shown in Figure 3.

**Figure 4:**
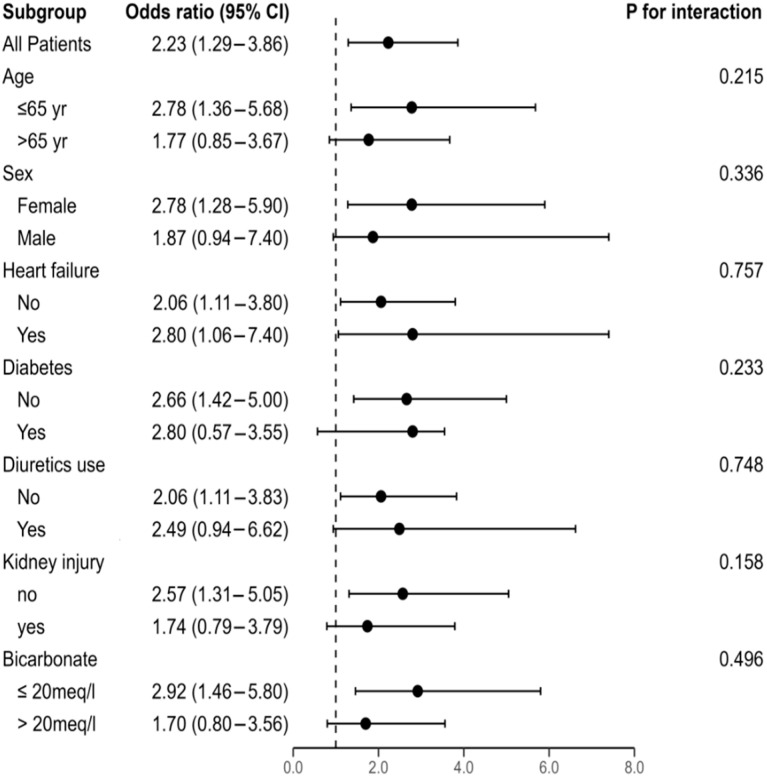
Subgroup analysis of the association between hypochloremia and hospital mortality. This forest plot presents the odds ratios (ORs) and corresponding 95% confidence intervals (CIs) for the association between hypochloremia and hospital mortality across various subgroups. Each point estimate (circle) indicates the OR for the respective subgroup, with horizontal lines representing the 95% CI. The vertical dashed line at OR = 1.0 represents no effect (null value). A point estimate to the right of this line suggests an increased hospital mortality, while a point estimate to the left suggests decreased odds. The *P* for interaction indicates whether the effect of hypochloremia and hospital mortality differs significantly across the subgroups shown. Subgroups include age (<65 vs. ≥65 years), sex, heart failure, diabetes, diuretic use, kidney injury, and bicarbonate (<20 vs. ≥20 meq/L).

Subgroup analysis, after adjusting for relevant covariates, showed that the association between hypochloremia and hospital mortality was stable and not modified in homogeneous dichotomized subgroups (all *p* > 0.1 for interactions). These results are presented in Figure 4. Missing Data are reported in Table S1 as a Supplementary Materials.

## 3. Discussion

The prevalence of dyschloremias in this study was high at 40.9%. Hypochloremia had a strong prognostic value, independent of natremia levels, in patients with acute medical conditions. It increased the risk of hospital mortality and prolonged the LOHS even after adjusting for relevant covariates. In contrast, there was no worsening of prognosis in hyperchloremic patients as judged by hospital mortality and LOHS. The frequency of dyschloremias in the literature can reach up to 40% of hospitalization cases [1,6,21]. The prevalence of hypochloremia in our study was higher (26.9%) than that found by Tani (8.8%) [2]. Huang found that 10.2% of the studied population suffered from hypochloremia in a cardiorenal improvement registry in China (*n* = 4762), and 20.1% suffered from hypochloremia in another registry of the Medical Information Mart for Intensive Care III (*n* = 3481) [14]. Jin X reported that 4593 (20.9%) patients in intensive care units suffered from hypochloremia at admission [16]. The prevalence of hyperchloremia at admission to the AMU in our study was 14% lower than that of hypochloremia. Ruan X et al. reported a higher prevalence of hyperchloremia compared to hypochloremia in patients with septic shock. They explained these results by the fact that their patients were resuscitated in the emergency department with chloride-rich solutions before their admission to the intensive care unit to restore their circulatory volume, leading to an iatrogenic chloride overload [21,22,23,24,25]. The prevalence of hyperchloremia can reach 25 to 45% in intensive care units [22]. Regarding the relationship between hypochloremia and mortality, several authors have reported that hypochloremia is an independent prognostic marker of mortality in both specific populations [9,10,11,12,13,14,15] and large populations [2,16,17]. The exact mechanisms underlying the relationship between hypochloremia and severe illnesses are not well understood, but three hypotheses can be proposed. First, hypochloremia might increase the release of cytokines [5], and the acceleration of inflammation is a well-known predictor of poor prognosis [14,26,27]. Second, hypochloremia might result from a homeostatic change related to an excess secretion of vasopressin, leading to increased water reabsorption [7,9,14]. Third, hypochloremia could be considered a marker of a complex association between severe pathologies, ultimately leading to a significantly increased risk of mortality [9,12,14]. Furthermore, chloride plays an important role in neutrophil function. Neutrophil phagosomes require the continuous passage of chloride through various chloride channels and cotransporters, providing substrates to myeloperoxidase to produce hypochlorous acid [21,28,29,30,31]. Low extracellular chloride concentrations have been associated with decreased neutrophil function [30,31], which could explain why patients with hypochloremia had a poorer prognosis. In our study, hypochloremia also had strong prognostic value independent from natremia. It is known that hyponatremia is a predictor of higher mortality and rehospitalization rates in the hospital [32,33]. Kondo T reported in a prospective study that in a model adjusted for hyponatremia, hypochloremia at admission and at discharge was still significantly associated with death from heart failure [5]. It is important to clarify the prognostic information of hypochloremia independently of sodium to consider hypochloremia as a treatment target. It is also necessary to note that severe hypo- and hypernatremia (e.g., natremia of 105 or 165 mmol/L) are often associated with critical illness and significant mortality risk. These extreme values, while infrequent, were included in our interaction model to allow visualization of the full spectrum of potential effects. However, clinical interpretation at these extremes should be performed with caution, as the physiological impact in real-world scenarios may differ from model-based predictions

Finally, in our study, the association of hypochloremia with mortality was stable across different dichotomized subgroups. These results are similar to those of other studies published in the literature [7,9,10,11,12,13].

Regarding hyperchloremia, it was not significantly associated with poor prognosis in this study, which is consistent with another study conducted on patients with septic shock that showed hyperchloremia was not associated with an increased risk of acute kidney injury or mortality [34]. Conversely, other observational studies have shown increased mortality in hyperchloremic patients who received chloride-rich solutions [35,36]. Sepsis and septic shock are among the leading causes of mortality in critically ill patients [37]. In our series, the majority of patients presented with sepsis upon admission to the emergency department and were likely given isotonic sodium chloride solutions, leading to an iatrogenic increase in their blood chloride levels. Patients in septic shock were transferred to the critical care unit, while borderline sepsis patients were transferred to our department. These patients responded to the prescribed treatments and vascular filling with saline solutions. This could partly explain why hyperchloremia was not associated with poor prognosis in our study. Hyperchloremia was also not associated with a longer length of stay. This is different from the results of another study where LOHS was significantly longer for patients with hyperchloremia. Those results were explained by the fact that resuscitating patients with renal or heart failure can lead to fluid overload, necessitating renal replacement therapy and thus longer hospital stays [17]. However, in our study, the number of patients with hyperchloremia was small, which could influence also our results.

### 3.1. Strengths of the Study

First, the study focused on a large and diverse population. Second, adjustments were made to minimize confounding biases. Third, subgroup analysis produced more robust results across different homogeneous dichotomized subgroups.

### 3.2. Limitations of the Study

This was a prospective, single-center study with potential selection and information biases. Strategies used to maintain adequate volume before AMU admission were not available and may have influenced the prevalence of dyschloremias in this study. Information on the precise cause of death was lacking, making it difficult to assess the relationship between dyschloremias and the specific cause of death. We only studied dyschloremias at admission and did not evaluate serum chloride levels during hospitalization and at discharge. Finally, data on the dosage and class of diuretics used were not available, so we could not clarify the causal relationship between dyschloremias and diuretic resistance.

### 3.3. Clinical Implications of the Findings

The study’s results may have clinical implications; first, chloride could help practitioners assess prognosis and closely monitor patients with acute medical conditions. Hypochloremia may be used in prognostic scores next to other parameters, especially in countries with limited access to resources. Our study employed a type 2 prognostic research approach focused on evaluating predictive factors—in this case, dyschloremia—rather than developing a comprehensive scoring system [38]. In our association study, we identified hypochloremia as a predefined exposure and examined its relationship with in-hospital mortality after adjusting for key covariates. Our findings suggest that hypochloremia could serve as a complementary marker within a broader risk stratification framework. We agree that future studies should investigate the incremental predictive benefit of including chloride levels in established scoring systems. Additionally, early preventive treatment of hypochloremia might have a beneficial effect on prognosis. Further studies are needed to explore these hypotheses.

## 4. Conclusions

In this study, the prevalence of dyschloremias in patients with acute medical conditions was high. Hypochloremia increased the risk of in-hospital mortality and prolonged LOHS, but hypercholoremia did not. It had a strong prognostic value independently of the serum sodium level, and its association with mortality was stable in the different dichotomized subgroups. Further studies are desirable in order to better judge the relationship between chloremia and the prognosis of patients with acute medical conditions.

## Figures and Tables

**Table 1:** Sociodemographics and clinical characteristics of the patients.

Characteristics	(*n* = 798)
Socio-demographic characteristics	
Age, years	57.3 ± 18.6
Age > 65 years	294 (36.8%)
Sex, male *n* (%)	414 (51.9%)
Comorbidities, *n* (%)	
Chronic diseases	509 (63.8%)
High blood pressure, mmhg	237 (29.7%)
Heart failure	149 (18.7%)
Diabetes mellitus	239 (29.9%)
Chronic kidney injury	68 (8.5%)
Cancer	28 (3.5%)
Diuretics use	138 (17.3%)
Clinical characteristics	
Charlson score	2 [0–3]
Mean arterial pressure, mmHg	87.4 ± 15.6
Heart rate, beats/minute	91 ± 18.3
Respiratory rate, cycles/minute	22.6 ± 5.6
Temperature, °C	37.3 ± 0.7

Data given as mean ± SD, or median [IQR] or number (%).

**Table 2:** Biological characteristics and the evolutionary criteria of the patients.

Characteristics	*n* = 798
Biological characteristics	
Chloride, mmol/L	101 ± 6.9
Sodium, mmol/L	136 ± 6.2
Bicarbonate, mmol/L	21.3 ± 5.7
Urea, mmol/L	6.6 [5–13.3]
Creatinine, µmol/L	81.3 [64.5–135.2]
Evolutionary criteria	
AMU length of stay, days	7 [4–11]
Hospital length of stay, days	8 [5–14]
AMU mortality, *n* (%)	82 (10.3%)
Hospital mortality, *n* (%)	102 (12.8%)

Data given as mean ± SD, or median [IQR] or number (%).

**Table 3:** Participant characteristics by serum chloride levels at admission: a comparative analysis with post-hoc testing.

Variables	Hypochloremia <98 mmol/L *n* = 215 (26.9%)	Normochloremia ≤98–106 mmol/L≤ *n* = 471 (59%)	Hyperchloremia >106 mmol/L *n* = 112 (14%)	*p*-Value
Demographic Data				
Age (mean ± SD) **	59.3 ± 17.1	56.5 ± 18.9	56.8 ± 19.9	0.183
Age > 65 years (%) *	38.6%	36.1%	36.6%	0.817
Male sex (%) *	54.9% ^b^	53.9%	37.5% ^b^	0.004
Comorbidities (%)				
Chronic diseases *	27.9% ^a^	39.5% ^a^	38.4%	0.012
Hypertension *	32.6%	28.3%	30.4%	0.521
Heart failure *	21.9%	18.7%	12.5%	0.119
Diabetes *	36.7% ^a^	27% ^a^	29.5%	0.034
Chronic kidney disease *	10.2%	7%	11.6%	0.168
Cance r *	1.9%	3%	8.9% ^c^	0.003
Diuretic use *	25.6% ^a,b^	14.6% ^a^	12.5% ^b^	<0.001
Clinical Parameters				
Charlson Score ***	2 [1–4] ^a^	2 [0–3] ^a^	2 [0–4]	0.049
Mean arterial pressure (mmHg) **	86.1 ± 15.5	88 ± 16	87.5 ± 14.4	0.370
Heart rate (bpm) **	93.1 ± 18.1	90 ± 17.2	91.1 ± 20.7	0.130
Respiratory rate (breaths/min) **	23.4 ± 5.6 ^b^	22.4 ± 5.5	21.7 ± 5.1 ^b^	0.014
Body temperature (°C) **	37.5 ± 0.8 ^b^	37.3 ± 0.6	37.2 ± 0.6 ^b^	0.001
Biological Parameters				
Chloride (mmol/L) **	92.3 ± 5.6 ^a,b^	101.9 ± 2.5 ^a,c^	110.1 ± 4.3 ^b,c^	<0.001
Sodium (mmol/L) **	131.2 ± 6.8 ^a,b^	136.5 ± 3.9 ^a,c^	141.1 ± 7.2 ^b,c^	<0.001
Bicarbonate (mmol/L) **	23.5 ± 6.9 ^a,b^	20.9 ± 4.7 ^a,c^	18.1 ± 5.3 ^b,c^	<0.001
Urea (mmol/L) ***	10 [5–19.9] ^a^	6.6 [3.3–11.6] ^a,c^	8.3 [5–16.6] ^c^	<0.001
Creatinine (µmol/L) ***	92 [69–169] ^a^	77 [62–119] ^a^	87 [65–173]	<0.001
Evolutionary Criteria				
AMU length of stay *** (days)	8 [5–13] ^b^	7 [4–11]	6 [4–10] ^b^	0.039
Hospital length of stay (days) ***	9 [6–17] ^b^	8 [5–13]	7 [4–13] ^b^	0.012
AMU mortality (%) *	16.7% ^(a,b)^	7.9% ^a^	8% ^b^	0.001
Hospital mortality (%) *	21.4% ^(a,b)^	10% ^a^	8% ^b^	<0.001

AMU: acute medical unit. * Data given as proportion of patients in each group (%). ** Data given as mean ± standard deviation. *** Data given as median [interquartile range]. ^a^ Significant difference between hypochloremia and normochloremia. ^b^ Significant difference between hypochloremia and hyperchloremia. ^c^ Significant difference between normochloremia and hyperchloremia.

**Table 4:** Logistic regression analysis of the association between serum chloride levels, in-hospital mortality, and hospital length of stay.

	In-Hospital Mortality	Hospital Length of Stay
	OR (95% CI) *p*-Value	β (95% CI) *p*-Value
Middle chloride level (98–106 mmol/L)	Ref	Ref
High chloride level (>106 mmol/L)	0.79 (0.35; 1.76) 0.570	−0.34 (−2.94; 2.27) 0.800
Low chloride level (<98 mmol/L)	2.23 (1.29; 3.86) 0.010	2.19 (−0.01; 4.39) 0.050

## Data Availability

Readers can access data to support the study’s findings. Data are available from the corresponding author upon reasonable request.

## Supplementary Materials

The following supporting information can be downloaded at: https://www.mdpi.com/article/10.3390/life15040676/s1, Table S1. Missing data.
