# Supplementary material for: Prevalence and Prognostic Significance of Chloride Levels in Patients with Acute Medical Conditions: A Prospective Observational Study
Source: Life (Basel). 2025 Apr 21;15(4):676. doi: 10.3390/life15040676 (PMC12029041; doi:10.3390/life15040676)
Supplement: Supplementary file 1 [file life-15-00676-s001.zip › life-3221599-supplementary.pdf]

Table S1. Missing data:.

Variables	*Missing data*
High blood pressure, mmhg	*1*
Heart rate, beats/minute	*1*
Respiratory rate, cycles/minute	*2*
Temperature, °c	*2*
Bicarbonate, mmol/L	*1*
